# Poly[[aqua­(μ-4,4′-bipyridyl-κ^2^ *N*:*N*′)bis­(μ-formato-κ^2^ *O*:*O*′)iron(II)] tetra­hydrate]

**DOI:** 10.1107/S1600536809034722

**Published:** 2009-09-09

**Authors:** Bin Jiang, Zhilu Liu

**Affiliations:** a Department of Pharmacy, Shandong Medical College, Jinan 250002, People’s Republic of China; b State Key Laboratory of Solid Lubrication, Lanzhou Institute of Chemical Physics, Chinese Academy of Sciences, Lanzhou 73000, People’s Republic of China

## Abstract

In the title compound, {[Fe(CHO_2_)_2_(C_10_H_8_N_2_)(H_2_O)]·4H_2_O}_*n*_, the Fe^II^ ion is coordinated by two 4,4′-bipyridyl (4,4′-bpy) ligands, three formate ligands and one water molecule. The slightly distorted octahedral FeN_2_O_4_ coordination results from the N atoms of two bridging 4,4′-bpy ligands, the O atoms of two bridging HCOO^−^ anions of *anti–anti* mode, all in *trans* positions around the metal centre, and the O atoms of one terminal HCOO^−^ anion and of one water molecule. The bridging formate ligands link the metal ions into chains that are further connected *via* 4,4′-bpy ligands into a framework structure. The three-dimensional structure is stabilized by extensive O—H⋯O hydrogen bonding. The crystals were twinned containing a 0.84:0.16 racemate.

## Related literature

For the potential applications of metal-organic frameworks, see: Jia *et al.* (2007 ▶); Hagrman *et al.* (1999 ▶); Kortz *et al.* (2003 ▶); Li *et al.* (1996 ▶); Liu *et al.* (2007 ▶); Seo *et al.* (2000 ▶); Wang *et al.* (2007 ▶); Yaghi *et al.* (1998 ▶).
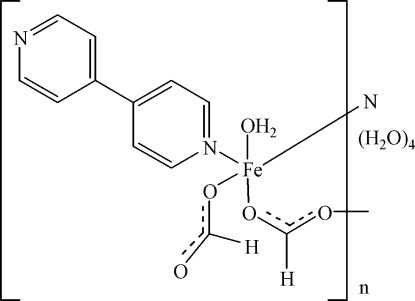


## Experimental

### 

#### Crystal data

[Fe(CHO_2_)_2_(C_10_H_8_N_2_)(H_2_O)]·4H_2_O
*M* *_r_* = 392.15
Monoclinic, 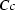

*a* = 10.5021 (6) Å
*b* = 20.1959 (11) Å
*c* = 8.1256 (4) Å
β = 102.367 (1)°
*V* = 1683.44 (16) Å^3^
*Z* = 4
Mo *K*α radiation
μ = 0.94 mm^−1^
*T* = 273 K
0.12 × 0.10 × 0.08 mm

#### Data collection

Bruker SMART CCD area-detector diffractometer
Absorption correction: multi-scan (*SADABS*; Bruker, 2005 ▶) *T*_min_ = 0.895, *T*_max_ = 0.928
4376 measured reflections
2523 independent reflections
2468 reflections with *I* > 2σ(*I*)
*R*_int_ = 0.031

#### Refinement

*R*[*F* ^2^ > 2σ(*F* ^2^)] = 0.034
*wR*(*F* ^2^) = 0.084
*S* = 1.00
2523 reflections
248 parameters
19 restraints
H atoms treated by a mixture of independent and constrained refinement
Δρ_max_ = 0.31 e Å^−3^
Δρ_min_ = −0.42 e Å^−3^
Absolute structure: Flack (1983 ▶), 1036 Friedel pairs
Flack parameter: 0.158 (18)

### 

Data collection: *SMART* (Bruker, 2005 ▶); cell refinement: *SAINT* (Bruker, 2005 ▶); data reduction: *SAINT*; program(s) used to solve structure: *SHELXS97* (Sheldrick, 2008 ▶); program(s) used to refine structure: *SHELXL97* (Sheldrick, 2008 ▶); molecular graphics: *SHELXTL* (Sheldrick, 2008 ▶); software used to prepare material for publication: *SHELXL97*.

## Supplementary Material

Crystal structure: contains datablocks global, I. DOI: 10.1107/S1600536809034722/pv2199sup1.cif

Structure factors: contains datablocks I. DOI: 10.1107/S1600536809034722/pv2199Isup2.hkl

Additional supplementary materials:  crystallographic information; 3D view; checkCIF report

## Figures and Tables

**Table 1:** Hydrogen-bond geometry (Å, °)

*D*—H⋯*A*	*D*—H	H⋯*A*	*D*⋯*A*	*D*—H⋯*A*
O5—H1*W*⋯O4^i^	0.82 (4)	1.97 (4)	2.693 (4)	146 (6)
O6—H3*W*⋯O3^ii^	0.82 (4)	1.98 (4)	2.792 (4)	173 (4)
O6—H4*W*⋯O9^iii^	0.82 (3)	1.93 (3)	2.753 (4)	175 (5)
O7—H5*W*⋯O8^iv^	0.82 (5)	2.22 (5)	3.028 (9)	171 (4)
O7—H6*W*⋯O4^ii^	0.82 (3)	2.46 (3)	3.117 (7)	137 (4)
O9—H10*W*⋯O1^iii^	0.82 (4)	2.16 (4)	2.954 (4)	165 (5)
O7—H6*W*⋯O2	0.82 (3)	2.61 (5)	3.158 (5)	125 (5)
O8—H7*W*⋯O7	0.82 (3)	1.94 (3)	2.763 (7)	174 (5)
O8—H8*W*⋯O6	0.82 (3)	2.031 (19)	2.797 (5)	155 (4)
O9—H9*W*⋯O8	0.82 (4)	1.99 (4)	2.779 (5)	163 (5)
O5—H2*W*⋯O6	0.82 (3)	1.94 (4)	2.729 (4)	161 (4)

Symmetry codes: (i) 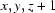; (ii) 
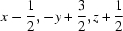
; (iii) 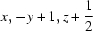; (iv) 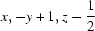.

**Fig. 1.**
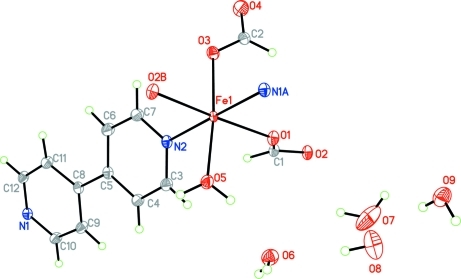
A view of the title compound with the atom-labeling scheme. Displacement ellipsoids are drawn at the 30% probability level. Symmetry codes for atoms: N1A, x+1/2, -y+3/2, z+1/2 and O2B, x+1/2, y-1/2, z.

**Fig. 2.**
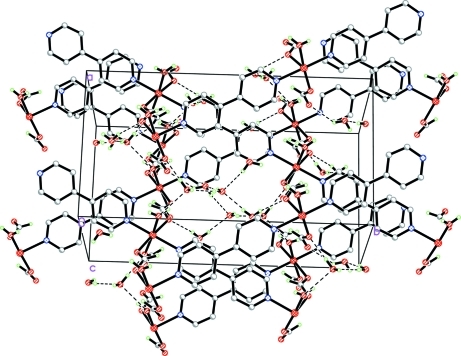
Packing diagram of the title compound showing hydrogen bonding; H-atoms not involved in H-bonds have been excluded for clarity.

## supplementary crystallographic information

### Comment

Design and construction of metal-organic frameworks (MOFs) have attracted considerable attention in recent years, not only for their intriguing structural motifs but also for their potential applications in the areas of catalysis, separation, gas absorption, molecular recognition, nonlinear optics, and magnetochemistry (Jia *et al.*, 2007; Li *et al.*, 1996; Seo *et al.*, 2000; Hagrman *et al.*, 1999; Yaghi *et al.*, 1998; Kortz *et al.*, 2003; Liu *et al.*, 2007; Wang *et al.*, 2007). A successful strategy for the design and synthesis of predictable MOFs is the assembly reaction between metal ions and well designed organic ligands. In this paper, we report the preparation and crystal structure of the title compound, (I).

The Fe^II^ ion in the title compound (Fig. 1) is octahedrally coordinated by two bridging 4,4'-bipyridyl (4,4'-bpy) ligands, two bridging HCOO^-^ (O1—C1—O2) groups in an anti-anti mode, all in *trans* positions around the metal ion, one terminal HCOO^-^ (O3—C2—O4), and one H_2_O molecule. The bridging formate ligands link metal ions to form chains running along the *ac* direction. The chain is further connected to other chains *via* 4,4'-bpy ligands. The three-dimensional structure is stabilized by extensive hydrogen bonding (Fig. 2 and Table 1).

### Experimental

The crystallization was performed in a 25 ml Teflon-lined stainless steel vessel. A mixture of 4,4'-bipyridyl ligand (1 mmol), iron(II) chloride tetrahydrate (1 mmol), and sodium formate (1 mmol) in 14 ml water was heated to 443 K, and kept at this temperature for one day. Green crystals were obtained after cooling to room temperature with the yield 75%.

### Refinement

The space group Cc was determined from successful refinement of the structure. However, an analysis of the data and a high value of Flack parameter indicated twinning which was resolved by applying an appropriate twin law and using 1031 Friedel pairs which were not merged. The BASF parameter was 0.1728, indicating a 0.83:0.17 racemate. All hydrogen atoms bound to carbon atoms were refined using a riding model with C—H = 0.93 Å and *U*_iso_(H) = 1.2U_eq_(C). The H-atoms of the water molecules are included in the refinement using the '*DFIX*' command with the H-atoms separated by 1.38 Å, and the H—O bonds were constrained to be 0.82 Å with error 0.01. An overall U_iso_ was allowed for all H-atoms of water molecules.

### Crystal data

**Table:**

[Fe(CHO_2_)_2_(C_10_H_8_N_2_)(H_2_O)]·4H_2_O	*F*(000) = 816
*M**_r_* = 392.15	*D*_x_ = 1.547 Mg m^−^^3^
Monoclinic, *C**c*	Mo *K*α radiation, λ = 0.71073 Å
Hall symbol: C -2yc	Cell parameters from 4008 reflections
*a* = 10.5021 (6) Å	θ = 2.2–28.3°
*b* = 20.1959 (11) Å	µ = 0.94 mm^−^^1^
*c* = 8.1256 (4) Å	*T* = 273 K
β = 102.367 (1)°	Block, green
*V* = 1683.44 (16) Å^3^	0.12 × 0.10 × 0.08 mm
*Z* = 4	

### Data collection

**Table:**

Bruker SMART CCD area-detector diffractometer	2523 independent reflections
Radiation source: fine-focus sealed tube	2468 reflections with *I* > 2σ(*I*)
graphite	*R*_int_ = 0.031
φ and ω scans	θ_max_ = 25.0°, θ_min_ = 2.0°
Absorption correction: multi-scan (*SADABS*; Bruker, 2005)	*h* = −12→11
*T*_min_ = 0.895, *T*_max_ = 0.928	*k* = −19→24
4376 measured reflections	*l* = −9→9

### Refinement

**Table:**

Refinement on *F*^2^	Secondary atom site location: difference Fourier map
Least-squares matrix: full	Hydrogen site location: inferred from neighbouring sites
*R*[*F*^2^ > 2σ(*F*^2^)] = 0.034	H atoms treated by a mixture of independent and constrained refinement
*wR*(*F*^2^) = 0.084	*w* = 1/[σ^2^(*F*_o_^2^) + (0.071*P*)^2^] where *P* = (*F*_o_^2^ + 2*F*_c_^2^)/3
*S* = 1.00	(Δ/σ)_max_ = 0.001
2523 reflections	Δρ_max_ = 0.31 e Å^−^^3^
248 parameters	Δρ_min_ = −0.42 e Å^−^^3^
19 restraints	Absolute structure: Flack (1983), 1036 Friedel pairs
Primary atom site location: structure-invariant direct methods	Flack parameter: 0.158 (18)

### Special details

**Table:**

Experimental. Elemental Analysis. Calc. for C_12_H_20_FeN_2_O_9_: C 36.73, H 5.10, N 12.24%; Found: C 36.65, H 5.02, N 12.14%.
Geometry. All e.s.d.'s (except the e.s.d. in the dihedral angle between two l.s. planes) are estimated using the full covariance matrix. The cell e.s.d.'s are taken into account individually in the estimation of e.s.d.'s in distances, angles and torsion angles; correlations between e.s.d.'s in cell parameters are only used when they are defined by crystal symmetry. An approximate (isotropic) treatment of cell e.s.d.'s is used for estimating e.s.d.'s involving l.s. planes.
Refinement. Refinement of *F*^2^ against ALL reflections. The weighted *R*-factor *wR* and goodness of fit *S* are based on *F*^2^, conventional *R*-factors *R* are based on *F*, with *F* set to zero for negative *F*^2^. The threshold expression of *F*^2^ > σ(*F*^2^) is used only for calculating *R*-factors(gt) *etc*. and is not relevant to the choice of reflections for refinement. *R*-factors based on *F*^2^ are statistically about twice as large as those based on *F*, and *R*- factors based on ALL data will be even larger.

### Fractional atomic coordinates and isotropic or equivalent isotropic displacement parameters (Å^2^)

**Table:**

	*x*	*y*	*z*	*U*_iso_*/*U*_eq_	
Fe1	0.99831 (11)	0.753902 (16)	0.64202 (13)	0.01649 (13)	
C1	0.7398 (3)	0.72764 (15)	0.4049 (4)	0.0274 (6)	
H1	0.7212	0.7713	0.4273	0.033*	
C2	1.0318 (4)	0.76341 (18)	0.2832 (5)	0.0339 (8)	
H2	0.9801	0.7256	0.2686	0.041*	
C3	0.7896 (3)	0.85353 (16)	0.6887 (5)	0.0342 (7)	
H3	0.7527	0.8158	0.7245	0.041*	
C4	0.7264 (3)	0.91325 (17)	0.6933 (4)	0.0338 (7)	
H4	0.6487	0.9149	0.7306	0.041*	
C5	0.7789 (3)	0.97064 (15)	0.6424 (4)	0.0294 (8)	
C6	0.8935 (4)	0.96383 (17)	0.5850 (5)	0.0401 (9)	
H6	0.9321	1.0007	0.5475	0.048*	
C7	0.9502 (4)	0.90224 (16)	0.5837 (5)	0.0381 (8)	
H7	1.0269	0.8989	0.5445	0.046*	
C8	0.7165 (3)	1.03639 (15)	0.6479 (4)	0.0280 (7)	
C9	0.5937 (3)	1.04287 (16)	0.6846 (5)	0.0359 (8)	
H9	0.5493	1.0055	0.7090	0.043*	
C10	0.5377 (3)	1.10457 (17)	0.6847 (5)	0.0355 (8)	
H10	0.4555	1.1076	0.7096	0.043*	
C11	0.7777 (4)	1.09450 (16)	0.6157 (5)	0.0355 (8)	
H11	0.8609	1.0932	0.5932	0.043*	
C12	0.7138 (3)	1.15402 (16)	0.6174 (4)	0.0327 (8)	
H12	0.7559	1.1923	0.5938	0.039*	
N1	0.5956 (3)	1.16024 (12)	0.6509 (3)	0.0276 (6)	
N2	0.9005 (3)	0.84703 (12)	0.6360 (3)	0.0267 (6)	
O1	0.8405 (2)	0.70271 (10)	0.4923 (3)	0.0311 (5)	
O2	0.6621 (2)	0.69888 (11)	0.2902 (3)	0.0305 (5)	
O3	1.0696 (2)	0.78393 (11)	0.4317 (3)	0.0330 (5)	
O4	1.0559 (3)	0.78812 (16)	0.1566 (4)	0.0569 (8)	
O5	0.9382 (3)	0.73151 (14)	0.8633 (3)	0.0370 (6)	
O6	0.7891 (3)	0.63516 (14)	0.9637 (4)	0.0482 (6)	
O7	0.6017 (4)	0.5754 (3)	0.4995 (8)	0.1160 (19)	
O8	0.7346 (5)	0.51513 (19)	0.7912 (5)	0.0972 (14)	
O9	0.8996 (3)	0.40816 (15)	0.7836 (4)	0.0561 (8)	
H1W	0.946 (5)	0.7580 (16)	0.942 (5)	0.080*	
H2W	0.892 (4)	0.6994 (13)	0.871 (5)	0.080*	
H3W	0.727 (3)	0.660 (2)	0.963 (5)	0.080*	
H4W	0.822 (5)	0.620 (2)	1.057 (3)	0.080*	
H5W	0.644 (5)	0.5542 (19)	0.444 (7)	0.080*	
H6W	0.626 (5)	0.6138 (10)	0.518 (7)	0.080*	
H7W	0.692 (4)	0.531 (2)	0.703 (3)	0.080*	
H8W	0.761 (5)	0.5426 (16)	0.865 (4)	0.080*	
H9W	0.850 (4)	0.4397 (15)	0.764 (6)	0.080*	
H10W	0.885 (5)	0.383 (2)	0.856 (5)	0.080*	

### Atomic displacement parameters (Å^2^)

**Table:**

	*U*^11^	*U*^22^	*U*^33^	*U*^12^	*U*^13^	*U*^23^
Fe1	0.0162 (2)	0.01436 (18)	0.0163 (2)	0.00272 (16)	−0.00227 (13)	−0.00061 (15)
C1	0.0252 (14)	0.0238 (13)	0.0303 (15)	−0.0001 (15)	−0.0001 (12)	0.0005 (15)
C2	0.0352 (19)	0.0367 (16)	0.030 (2)	−0.0028 (15)	0.0070 (15)	−0.0049 (15)
C3	0.037 (2)	0.0232 (16)	0.0437 (18)	0.0014 (14)	0.0122 (15)	0.0034 (13)
C4	0.0307 (19)	0.0267 (17)	0.045 (2)	0.0046 (13)	0.0110 (14)	0.0010 (13)
C5	0.028 (2)	0.0250 (16)	0.0321 (17)	0.0044 (12)	0.0001 (14)	−0.0016 (12)
C6	0.040 (2)	0.0225 (17)	0.061 (2)	0.0034 (13)	0.0188 (18)	0.0044 (15)
C7	0.0352 (19)	0.0268 (16)	0.056 (2)	0.0049 (13)	0.0171 (15)	−0.0004 (15)
C8	0.032 (2)	0.0226 (15)	0.0276 (16)	0.0048 (12)	0.0014 (14)	0.0007 (12)
C9	0.0319 (18)	0.0238 (16)	0.053 (2)	0.0017 (12)	0.0111 (16)	0.0050 (14)
C10	0.0259 (18)	0.0288 (16)	0.053 (2)	0.0048 (12)	0.0121 (15)	0.0011 (14)
C11	0.0274 (18)	0.0280 (17)	0.051 (2)	0.0042 (13)	0.0073 (15)	0.0010 (14)
C12	0.0303 (18)	0.0231 (16)	0.0438 (19)	0.0005 (12)	0.0059 (15)	0.0011 (13)
N1	0.0275 (13)	0.0231 (13)	0.0300 (13)	0.0046 (10)	0.0009 (11)	0.0000 (10)
N2	0.0266 (14)	0.0217 (13)	0.0295 (13)	0.0040 (10)	0.0005 (11)	−0.0010 (10)
O1	0.0256 (12)	0.0278 (11)	0.0339 (12)	0.0014 (9)	−0.0067 (10)	−0.0007 (9)
O2	0.0278 (11)	0.0285 (12)	0.0290 (12)	0.0007 (9)	−0.0079 (10)	−0.0037 (9)
O3	0.0379 (13)	0.0348 (13)	0.0252 (12)	−0.0003 (10)	0.0040 (10)	−0.0025 (9)
O4	0.0724 (19)	0.070 (2)	0.0291 (13)	−0.0197 (15)	0.0119 (12)	−0.0044 (13)
O5	0.0461 (16)	0.0362 (13)	0.0292 (13)	−0.0115 (12)	0.0090 (11)	−0.0025 (11)
O6	0.0475 (15)	0.0446 (15)	0.0538 (17)	0.0017 (11)	0.0134 (12)	0.0059 (12)
O7	0.089 (3)	0.092 (3)	0.161 (6)	−0.021 (3)	0.013 (3)	0.057 (3)
O8	0.124 (4)	0.068 (3)	0.086 (3)	0.017 (2)	−0.008 (2)	−0.020 (2)
O9	0.063 (2)	0.0502 (18)	0.0540 (18)	−0.0072 (14)	0.0096 (15)	0.0084 (13)

### Geometric parameters (Å, °)

**Table:**

Fe1—O5	2.079 (3)	C8—C9	1.390 (5)
Fe1—O3	2.097 (3)	C8—C11	1.390 (5)
Fe1—O1	2.105 (2)	C9—C10	1.378 (5)
Fe1—O2^i^	2.105 (2)	C9—H9	0.9300
Fe1—N2	2.139 (3)	C10—N1	1.334 (4)
Fe1—N1^ii^	2.144 (3)	C10—H10	0.9300
C1—O1	1.247 (4)	C11—C12	1.378 (5)
C1—O2	1.243 (4)	C11—H11	0.9300
C1—H1	0.9300	C12—N1	1.333 (5)
C2—O4	1.218 (5)	C12—H12	0.9300
C2—O3	1.257 (4)	N1—Fe1^iii^	2.144 (3)
C2—H2	0.9300	O2—Fe1^iv^	2.105 (2)
C3—N2	1.330 (4)	O5—H1W	0.82 (4)
C3—C4	1.381 (5)	O5—H2W	0.82 (3)
C3—H3	0.9300	O6—H3W	0.82 (4)
C4—C5	1.384 (5)	O6—H4W	0.82 (3)
C4—H4	0.9300	O7—H5W	0.82 (5)
C5—C6	1.388 (5)	O7—H6W	0.82 (3)
C5—C8	1.486 (4)	O8—H7W	0.82 (3)
C6—C7	1.380 (5)	O8—H8W	0.82 (3)
C6—H6	0.9300	O9—H9W	0.82 (4)
C7—N2	1.338 (4)	O9—H10W	0.82 (4)
C7—H7	0.9300		
			
O5—Fe1—O3	174.46 (10)	N2—C7—H7	118.3
O5—Fe1—O1	92.60 (10)	C6—C7—H7	118.3
O3—Fe1—O1	92.62 (10)	C9—C8—C11	116.7 (3)
O5—Fe1—O2^i^	88.06 (9)	C9—C8—C5	121.7 (3)
O3—Fe1—O2^i^	86.81 (9)	C11—C8—C5	121.6 (3)
O1—Fe1—O2^i^	177.17 (10)	C10—C9—C8	120.0 (3)
O5—Fe1—N2	88.72 (11)	C10—C9—H9	120.0
O3—Fe1—N2	88.90 (10)	C8—C9—H9	120.0
O1—Fe1—N2	95.96 (9)	N1—C10—C9	123.3 (3)
O2^i^—Fe1—N2	86.81 (10)	N1—C10—H10	118.4
O5—Fe1—N1^ii^	90.57 (11)	C9—C10—H10	118.4
O3—Fe1—N1^ii^	91.81 (10)	C12—C11—C8	119.3 (3)
O1—Fe1—N1^ii^	84.07 (9)	C12—C11—H11	120.4
O2^i^—Fe1—N1^ii^	93.18 (10)	C8—C11—H11	120.4
N2—Fe1—N1^ii^	179.28 (13)	N1—C12—C11	124.1 (3)
O1—C1—O2	125.4 (3)	N1—C12—H12	117.9
O1—C1—H1	117.3	C11—C12—H12	117.9
O2—C1—H1	117.3	C12—N1—C10	116.6 (3)
O4—C2—O3	126.6 (4)	C12—N1—Fe1^iii^	122.3 (2)
O4—C2—H2	116.7	C10—N1—Fe1^iii^	121.0 (2)
O3—C2—H2	116.7	C3—N2—C7	116.6 (3)
N2—C3—C4	123.6 (3)	C3—N2—Fe1	121.9 (2)
N2—C3—H3	118.2	C7—N2—Fe1	121.4 (2)
C4—C3—H3	118.2	C1—O1—Fe1	126.7 (2)
C3—C4—C5	119.9 (3)	C1—O2—Fe1^iv^	122.8 (2)
C3—C4—H4	120.1	C2—O3—Fe1	126.3 (2)
C5—C4—H4	120.1	Fe1—O5—H1W	122 (3)
C4—C5—C6	116.6 (3)	Fe1—O5—H2W	122 (3)
C4—C5—C8	122.2 (3)	H1W—O5—H2W	115 (4)
C6—C5—C8	121.3 (3)	H3W—O6—H4W	114 (4)
C7—C6—C5	119.9 (3)	H5W—O7—H6W	114 (5)
C7—C6—H6	120.1	H7W—O8—H8W	114 (4)
C5—C6—H6	120.1	H9W—O9—H10W	115 (5)
N2—C7—C6	123.4 (3)		

Symmetry codes: (i) *x*+1/2, −*y*+3/2, *z*+1/2; (ii) *x*+1/2, *y*−1/2, *z*; (iii) *x*−1/2, *y*+1/2, *z*; (iv) *x*−1/2, −*y*+3/2, *z*−1/2.

### Hydrogen-bond geometry (Å, °)

**Table:**

*D*—H···*A*	*D*—H	H···*A*	*D*···*A*	*D*—H···*A*
O5—H1W···O4^v^	0.82 (4)	1.97 (4)	2.693 (4)	146 (6)
O6—H3W···O3^vi^	0.82 (4)	1.98 (4)	2.792 (4)	173 (4)
O6—H4W···O9^vii^	0.82 (3)	1.93 (3)	2.753 (4)	175 (5)
O7—H5W···O8^viii^	0.82 (5)	2.22 (5)	3.028 (9)	171 (4)
O7—H6W···O4^vi^	0.82 (3)	2.46 (3)	3.117 (7)	137 (4)
O9—H10W···O1^vii^	0.82 (4)	2.16 (4)	2.954 (4)	165 (5)
O7—H6W···O2	0.82 (3)	2.61 (5)	3.158 (5)	125 (5)
O8—H7W···O7	0.82 (3)	1.94 (3)	2.763 (7)	174 (5)
O8—H8W···O6	0.82 (3)	2.03 (2)	2.797 (5)	155 (4)
O9—H9W···O8	0.82 (4)	1.99 (4)	2.779 (5)	163 (5)
O5—H2W···O6	0.82 (3)	1.94 (4)	2.729 (4)	161 (4)

Symmetry codes: (v) *x*, *y*, *z*+1; (vi) *x*−1/2, −*y*+3/2, *z*+1/2; (vii) *x*, −*y*+1, *z*+1/2; (viii) *x*, −*y*+1, *z*−1/2.
